# Role of sclerostin in mastocytosis bone disease

**DOI:** 10.1038/s41598-024-83851-0

**Published:** 2025-01-02

**Authors:** Aneta Szudy-Szczyrek, Radosław Mlak, Dominika Pigoń-Zając, Witold Krupski, Marcin Mazurek, Aleksandra Tomczak, Karolina Chromik, Aleksandra Górska, Paweł Koźlik, Adrian Juda, Anna Kokoć, Maciej Dubaj, Tomasz Sacha, Marek Niedoszytko, Grzegorz Helbig, Michał Szczyrek, Justyna Szumiło, Teresa Małecka-Massalska, Marek Hus

**Affiliations:** 1 Department of Haematooncology and Bone Marrow Transplantation, Medical University of Lublin, Lublin, Staszica Street 11, 20-081 Poland; 2 Department of Laboratory Diagnostics, Medical University of Lublin, Lublin, Poland; 3 Department of Human Physiology, Medical University of Lublin, Lublin, Poland; 4 Department of Medical Radiology, Medical University of Lublin, Lublin, Poland; 5 Department of Hematology and Bone Marrow Transplantation, Medical University of Silesia in Katowice, Katowice, Poland; 6 Department of Pulmonology and Allergology, Medical University of Gdańsk, Gdańsk, Poland; 7 Chair of Hematology, Jagiellonian University, Kraków, Poland; 8 Department of Pneumonology, Oncology and Allergology, Medical University of Lublin, Lublin, Poland; 9 Department of Clinical Pathomorphology, Medical University of Lublin, Lublin, Poland

**Keywords:** Bone remodeling, Bones, Mastocytosis, Osteolysis, Osteosclerosis, Sclerostin, Haematological cancer, Biomarkers

## Abstract

Mastocytosis is a heterogeneous group of disorders, characterized by accumulation of clonal mast cells which can infiltrate several organs, most often spine (70%). The pathogenesis of mastocytosis bone disease is poorly understood. The main aim of the study was to investigate whether neoplastic mast cells may be the source of sclerostin and whether there is an association between sclerostin and selected bone remodeling markers with mastocytosis related bone disease. We assessed sclerostin, bioactive sclerostin, and *SOST* gene expression in HMC-1.2 human mast cell culture supernatants and plasma of SM patients (*n* = 39). We showed that human mast cells can secrete sclerostin, and after their stimulation with IL-6, there is a significant increase in *SOST* gene expression. We observed significantly higher levels of sclerostin in patients diagnosed with more advanced disease. We observed a statistically significant correlation between concentations of sclerostin and its bioactive form and the concentration of alkaline phosphatase (ALP), and between sclerostin and interleukin-6 (IL-6). We observed that significantly higher sclerostin concentrations are present in patients with increased sclerosis of the spongy bone. Sclerostin may serve as a marker of more advanced disease and bone disease in mastocytosis. Further studies are justified to evaluate its role in mastocytosis.

## Introduction

Mastocytosis includes a group of neoplastic diseases characterized by excessive proliferation and accumulation of pathological mast cells in one or more organs. The clinical picture varies widely, from a disease limited to the skin - cutaneous mastocytosis (CM), to involvement of multiple organs - systemic mastocytosis (SM)^1,2^. Mastocytosis is a rare (orphan) disease. Most epidemiological data on the disease comes from the Scandinavian countries of Denmark, Sweden and Norway, due to the prevalence in these nations. Its average annual incidence, according to various researches, ranges from 0.77 to 2.77 per 100,000 inhabitants, and the prevalence varies from 9.59 to 17.2 per 100,000 inhabitants^3–7^. These rates are rising every year, mainly due to increased awareness and proper diagnosis of the disease. There are no significant differences in prevalence between the sexes, there is only a predilection among Caucasians^8^. SM is more often observed among the elderly (average age is 60), although it also occurs in the pediatric population^8^. Mast cells originate from hematopoietic stem cells; their progenitor cells express CD13, CD34 and CD117 (c-kit) and are detectable in bone marrow and peripheral blood. They differ from other cells of the granulocytic lineage by unique phenotypic and functional features, producing significant amounts of histamine, heparin and other mediators, and expressing the surface IgE receptor^1,2^.

Bone involvement occurs in about 70% of patients with SM. The most common findings are osteopenia and osteoporosis. Osteoporotic fractures occur in about 40% of patients. The cases described radiologically include mixed generalized bone remodeling, osteosclerotic and osteolytic focal lesions located in the spine and long bones, and compression fractures. The pathogenesis of bone alterations in SM remains unclear. Bone infiltration by mast cells and the influence of inflammatory mediators are considered. Histamine, tryptase and heparin may directly activate osteoclasts. Patients also are observed to have increased levels of cytokines involved in bone remodeling: IL-1 (interleukin 1), IL-6, TNF-α (tumor necrosis factor alpha) and RANKL (receptor activator of nuclear factor kappa-B ligand)^9–12^.

Sclerostin is a bone turnover protein that plays a crucial role in reducing osteoblastic bone formation by inhibiting the Wnt signaling pathway^13,14^. It is commonly thought of as an osteocyte-specific protein, although increased mRNA expression for sclerostin has been reported in cells of many other tissues and organs - cartilage, kidney, heart and liver. Interestingly, increased expression of the *SOST* gene encoding sclerostin and elevated levels of this protein are found in cell lines and cells isolated from patients with primary and metastatic bone cancers, including osteosarcoma, chondrosarcoma, multiple myeloma, as well as breast and prostate cancer^15–17^. Reports indicating elevated levels of sclerostin in the serum of patients with SM suggest the importance of studying the function of this protein in the pathogenesis of bone alterations in SM^18^.

The main aim of the following study was to evaluate the secretion of sclerostin by mast cells in vitro and to relate its levels in SM patients to selected clinical features and levels of other bone remodeling markers.

## Methods

### The study population

The study group included 39 patients with a diagnosis of mastocytosis, among them 13 with aggressive systemic mastocytosis (ASM), 1 patient with systemic mastocytosis with associated hematologic malignancy (SM-AHN), 18 with indolent systemic mastocytosis (ISM), 4 with smoldering systemic mastocytosis (SSM) and 3 with cutaneous mastocytosis (CM). Patients were enrolled in the study at the Department of Hematooncology and Bone Marrow Transplantation in Lublin, the Department of Hematology and Bone Marrow Transplantation in Katowice, the Department of Pulmonology and Allergology in Gdansk, and the Department of Hematology at Jagiellonian University in Krakow between 2019 and 2022. The diagnosis of mastocytosis was made based on a biopsy of the affected organ (bone marrow trepanation biopsy, biopsy of involved skin), according to the 2022 WHO classification^19^. The specimens tested were approximately 2.5 ml of peripheral blood collected into plasma gel tubes. Samples were centrifuged at 3,000 rpm for 10 min, the plasma was pipetted into tubes and stored at -80 °C until further analysis. Blood was drawn before starting treatment and in patients with ASM in progression after confirmation of progression, at least 14 days after completion of previous treatment. The 8 patients with ASM progression were treated with different cytoreductive therapies (cladribine [*n* = 4], midostaurin [*n* = 2]: hydroxycarbamide [*n* = 10], imatinib [*n* = 1]), due to the small group, these data were not included in the statistical analysis. The whole-body low-dose bone computed tomography was used to assess the severity of bone disease in patients with SM. The study was performed at the II Department of Medical Radiology in Lublin using a 64-slice General Electric Light Speed VCT scanner (GE Medical System, Milwaukee, WI, USA).

### Cell line culture

The HMC-1.2 cell line was obtained from the EMD Millipore (Cat. No. SCC062, EMD Millipore; Burlington, Massachusetts, USA) and was cultured according to the manufacturer’s protocol in IMDM culture medium (Cat. No. P04-20150, PAN BIOTECH; Aidenbach, Bayern, Germany), supplemented with 10% heat-inactivated fetal bovine serum (FBS, Cat. No. ES-009-B, EMD Millipore; Burlington, Massachusetts, USA), 1X penicillin/streptomycin (Cat. No. TMS-AB2-C, EMD Millipore; Burlington, Massachusetts, USA) and 1.2 mM α-thioglycerol (Cat. No. M6145-100 mL, Sigma-Aldrich; Saint Louis, Missouri, USA). Culture was held at 37 °C in a humidified atmosphere containing 95% air and 5% CO_2_. HMC-1.2 cells were passaged 2–3 times a week, when the cell density was at 1 × 10^6^- 1.5 × 10^6^ cells/mL and typically plated at a density of 2.5 × 10^5^ cells/mL. After reaching the appropriate culture density, human mast cells were transferred to a 96-well plate (2 × 10^5^ cells/200 µL) in triplicate to undergo stimulation with IL-6 (Cat. No. 259381, Abcam; Cambridge, UK), which induces differentiation and degranulation of mast cells and GM-CSF (granulocyte macrophage colony-stimulating factor) (Cat. No. 259367, Abcam; Cambridge, UK). The available literature shows that in patients with mastocytosis who experience bone degradation, IL-6 is significantly higher compared to those with mastocytosis and no bone changes^20^. Thus, in our experiment, HMC-1.2 cell line demonstrating persistent IL-6 production^21^ was supplemented with additional IL-6 to ensure that we would obtain conditions typical for the initiation of unfavorable changes in bone tissue, i.e., increased inflammation. The experiment used concentrations of 10, 50 and 100 ng/mL IL-6 and a concentration of 100 ng/mL GM-CSF compared to unstimulated HMC-1.2 cell line (control). The tested cells and supernatants were collected after 6, 24 and 48 h of incubation for further analysis. To eliminate the potential influence of the presence of FBS on the measurement of sclerostin concentration in an unstimulated mast cell culture, we subtracted the absorbance generated by the medium with FBS from the absorbance obtained in the sample with an unstimulated cell line.

### Sclerostin measurements

The evaluation of sclerostin (ELISA BI-20492 from Biomedica, Vienna, Austria) and bioactive sclerostin (ELISA BI-20472 from Biomedica, Vienna, Austria) concentrations was performed in the sera of patients as well as in cell culture supernatants. A standard enzyme-linked immunosorbent assay (ELISA) was used to determine the exact concentration according to the manufacturer’s protocol. All samples were analyzed in triplicate during three consecutive sessions, and the average values were recorded.

### *SOST* gene expression

Total RNA was extracted from cell cultures using the RNeasy Mini Kit (Qiagen, Canada). For the preparation of cDNA, the quality and concentration of the obtained material were then assessed spectrophotometrically (NanoDrop 2000c/2000, ThermoFisher Scientific) and reverse transcription was performed to obtain cDNA (High Capacity cDNA Reverse Transcription Kit, ThermoFisher Scientific, Waltham, MA, USA). For the preparation of the reaction mixture, TaqMan Universal MasterMix (ThermoFisher Scientific, Waltham, MA, USA), SOST gene probe (Assay ID: Hs00228830_m1, ThermoFisher Scientific, Waltham, MA, USA) and ACTB control gene probe (Assay ID: Hs03023943_g1, ThermoFisher Scientific, Waltham, MA, USA) were used. The reaction was performed using the StepOnePlus Real-Time PCR System (Applied Biosystems), and data analysis was conducted using QuantStudio software (Applied Biosystems). Each sample was analyzed in triplicate. The relative level of SOST gene expression was calculated based on normalization to ACTB as a housekeeping gene using the 2^−^ΔΔCt method.

### Statistical analysis

Statistical analysis was conducted using MedCalc 15.8 software (MedCalc statistical software, Belgium). Quantitative variables derived from cultures were compared using parametric t-tests (data distribution was represented using mean and standard deviation). Categorical variables were expressed using absolute numbers and percentages. However, due to non-normal distribution (assessed using the D’Agostino-Pearson test) in the analysis of continuous data from patients, non-parametric tests, median, and interquartile ranges (IQR) were utilized as measures of central tendency and dispersion. Mann-Whitney U test (for comparisons between 2 groups) or ANOVA Kruskal-Wallis test (for comparisons between more than 2 groups) were used to assess quantitative variables (sclerostin concetration, bioactive sclerostin concentration, *SOST* expression), depending on selected categorical demographic and clinical variables, including bone variables (form of mastocytosis, splenomegaly, level of tryptase > 20ug/l, presence of bone sclerosis). Spearman’s rank correlation test was employed to evaluate the correlation between selected quantitative variables (concentration of sclerostin, bioactive sclerostin, IL-6 and ALP activity). In all tests, results with *p* < 0.05 were interpreted as statistically significant.

## Results

### Patient characteristics

In the study population, there were slightly more women (56.4%). The median age was 49 years (range 17–76 years). The most common diagnosis was ISM (46.2%), followed by ASM in progression (20.5%), ASM *de novo* (12.8%), SMM (10.3%), CM (7.7%), and SM-AHN (2.6%). The majority of patients were in very good performance status (ECOG = 0). Hepatomegaly, splenomegaly, and lymphadenopathy were observed in 23.1%, 41%, and 10.3% of patients, respectively. Bone lesions (fractures, mainly of the spine) were noted in 17.9% of patients. In the analyzed group, 85% of patients were carriers of the KIT D816V mutation, in one patient the coexisting KIT D816V and D816H mutations were detected. Most patients were treated using antihistamines (48.7%), bisphosphonates (25.6%), and glucocorticosteroids (7.7%). (Table 1).

**Table 1:** Characteristics of the patients in the study group.

Variable	*n* = 39 (100%)
Sex	
Women	22 (56.4)
Men	17 (43.6)
Age [years]	
Median (min-max)	49 (17–76)
< 65	29 (74.4%)
≥ 65	10 (25.6%)
Diagnosis	
ASM *de novo*	5 (12.8%)
ASM in progression	8 (20.5%)
SM-AHN	1 (2.6%)
SMM	4 (10.3%)
ISM	18 (46.2%)
CM	3 (7.7%)
Tryptase > 20.0 µg/l** (normal range: 5–11,4 µg/l)	28 (82.4%)
No data: [*n* = 5]	
ECOG	
0	24 (63.2%)
1	5 (13.2%)
2	8 (21.1%)
3	1 (2.6%)
Hepatomegaly [n (%)]	9 (23.1%)
Splenomegaly [n (%)]	16 (41.0%)
Lymphadenopathy [n (%)]	4 (10.3%)
Ascites [n (%)]	2 (5.1%)
Bone lesions (fractures) [n (%)]	7 (17.9%)
Phosphorus [mmol/l] [median (min-max)]	1.13 (0.28–2.39)
Calcium [mmol/l] [median (min-max)]	2.39 (1.72–10.10)
*KIT* mutation [n (%)]	23 (85%) *No data: [n = 12]*
Comorbidities [n (%)]:	32 (82%)
Gastrointestinal complaints	17 (43.6%)
Circulatory system complaints	16 (41%)
Anaphylaxis	12 (30.8%)
Cancer/precancerous conditions	6 (15.4%)
Diabetes	2 (5.1%)
Treatment [n (%)]	
Antihistamines	19 (48.7%)
Bisphosphonates	10 (25.6%)
Glucocorticosteroids	3 (7.7%)

*ASM* aggressive systemic mastocytosis, *CM* cutaneous mastocytosis, *ISM* indolent systemic mastocytosis, *SM-AHN* systemic mastocytosis with associated hematological neoplasm, *SSM* smouldering systemic mastocytosis.

*statistically significant results.

**serum tryptase > 20 µg/L is a diagnostic criterion for SM^14^.

### Sclerostin secretion and *SOST* gene expression in HMC-1.2 cell line

**Fig. 1:**
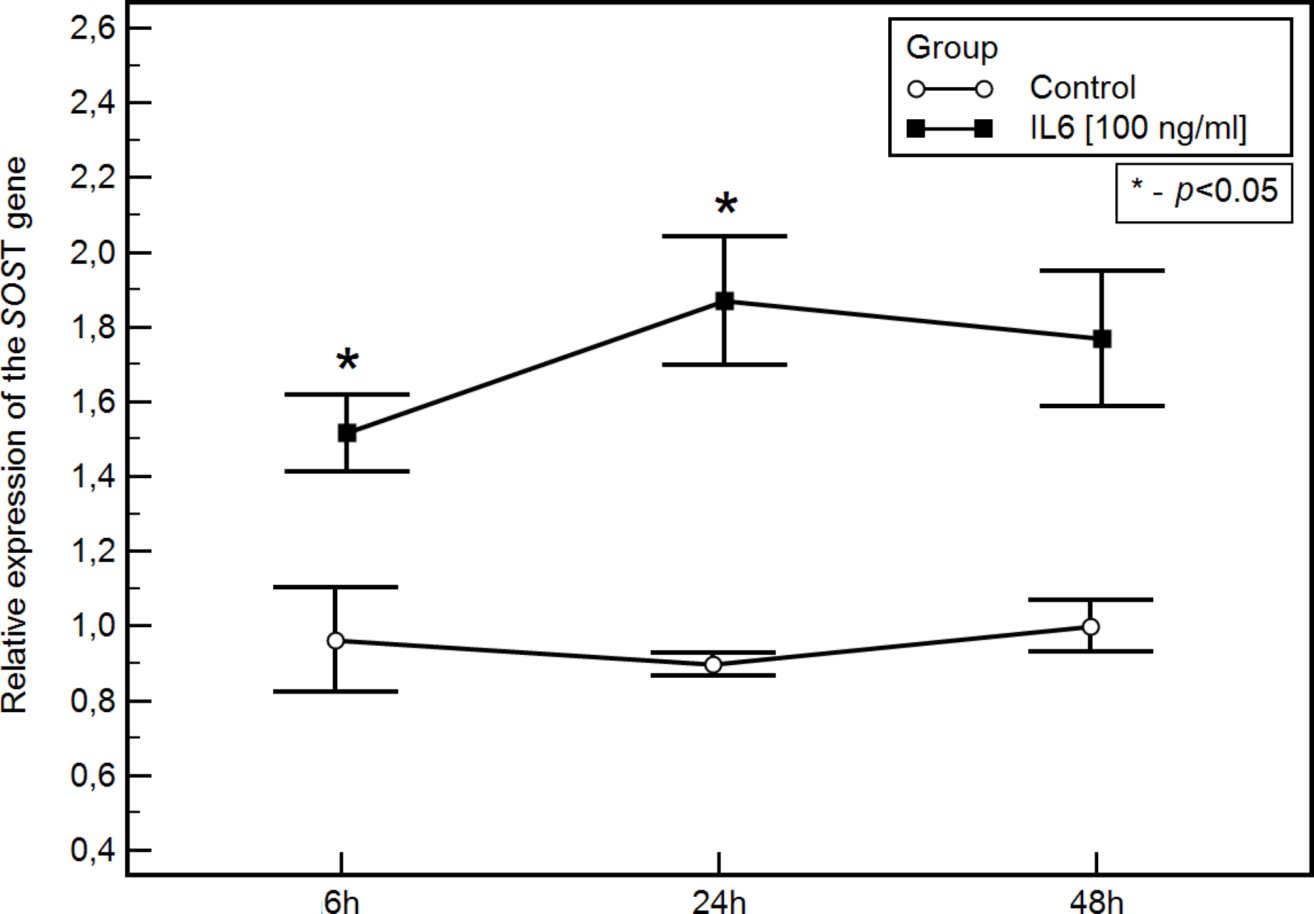
Relative SOST gene expression (mean ± SD) in the HMC1.2 cell line at 6, 24 and 48 h after IL-6 stimulation compared to the control group - the same cell line not subjected to IL-6 stimulation. * - statistically significant values (*p* < 0.05).

**Fig. 2:**
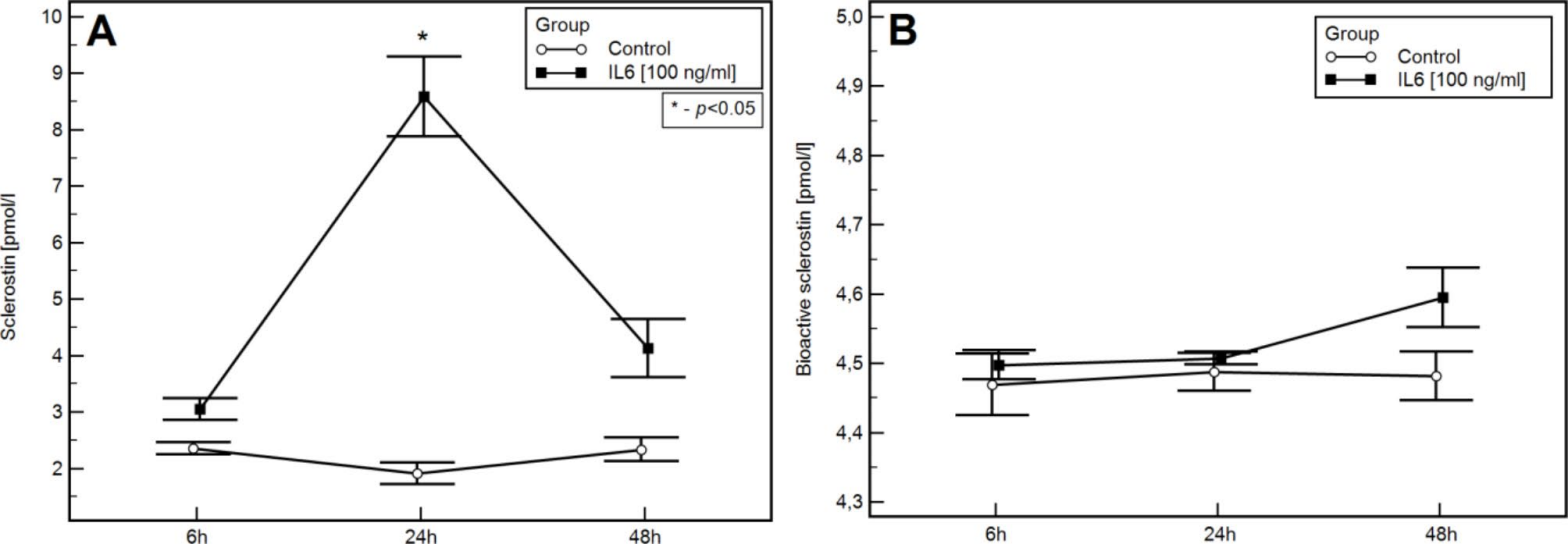
The concentration of sclerostin (mean ± SD)[pmol/l] (**a**) and its bioactive form (mean ± SD)[pmol/l] (**b**) in the supernatants from the HMC1.2 cell line after 6, 24, and 48 h after stimulation with IL-6 compared to the control group - the same cell line not subjected to IL-6 stimulation. * - statistically significant values (*p* < 0.05).

Based on studies on the HMC-1.2 cell line, we found that unstimulated, neoplastically transformed mast cells are capable of secreting sclerostin. Furthermore, upon stimulation with IL-6, there is an increase in the expression level of the *SOST* gene and sclerostin protein. In the culture stimulated with IL-6 (at a concentration of 100 ng/ml), compared to the control, we observed a significant increase in SOST gene expression at 6 h (mean ± SD, respectively: 1.5 ± 0.2 vs. 1 ± 0.2; Figs. 1) and 24 h (mean ± SD, respectively: 1.9 ± 0.3 vs. 8.6 ± 1.2; Fig. 1) post-stimulation, as well as a significant increase in sclerostin protein (mean ± SD, respectively: 0.9 ± 0.05 vs. 1.9 ± 0.3 [pmol/l]; Fig. 2A) 24 h post-stimulation. However, no significant differenceregarding bioactive sclerostin was noted in the assessed time points (Fig. 2B).

### Comparison of plasma concentration of sclerostin depending on demographic and clinical variables

We observed significantly higher sclerostin concentrations in the plasma of patients with: ASM diagnosed *de novo* vs. in progression (22 vs. 40.9 pmol/l), ASM in progression vs. SMM or CM (40.9 vs. 32.1 or 19.3 [pmol/l]), SM-AHN vs. SMM or CM (55.9 vs. 32.1 or 19.3 [pmol/l]), and SSM vs. ISM (32.1 vs. 18.7 [pmol/l]). Additionally, we observed significantly higher sclerostin concentrations in the plasma of patients with a diagnosis of ASM (both *de novo* and in progression), SM-AHN, and SSM compared to patients with less advanced mastocytosis: ISM and CM (34.2 vs. 19.3 [pmol/l]). Furthermore, significantly higher sclerostin concentrations were observed in patients with tryptase concentration > 20 µg/l (30.70 vs. 16.92 [pmol/l]). We also observed significantly higher sclerostin concentrations in patients with splenomegaly (34.2 vs. 19.7 [pmol/l]) (Table 2).

**Table 2:** Association between sclerostin [pmol/l] and bioactive sclerostin [pmol/l] concentrations and selected demographic and clinical variables (only statistically significant results are presented).

Variable	Sclerostin [pmol/l]		Bioactive sclerostin [pmol/l]	
	Median[IQR]	*p*	Median[IQR]	***p***
Diagnosis				
ASM *de novo* ASM in progression SM-AHN SSM ISM CM	22.0 [11.4–37.8] 40.9 [30.9–50.0] 55.9 [55.9–55.9] 32.1 [29.5–55.1] 18.7 [11.9–22.5] 19.3 [16.9–23.8]	0.0112*	50.8 [21.5–70.7] 64.4 [38.3-105.3] 65.3 [65.3–65.3] 89.9 [52.9–200.0] 43.2 [33.0-59.2] 71.0 [50.8–76.8]	0.3903
Diagnosis				
ASM *de novo*, ASM in progression, SM-AHN, SSM ISM, CM	34.2 [28.1–46.9] 19.3 [14.0-22.5]	0.0013*	65.0 [34.0-100.8] 44.1 [33.0-64.3]	0.1763
Tryptase > 20.0 µg/l** (normal range: 5–11,4 µg/l)				
No Yes *No data [n = 5]*	16.9 [11.9–19.7] 32.1 [20.7–42.3]	0.0188*	43.2 [33.0-44.1] 59 [33.4–92.1]	0.2225
Splenomegaly				
No Yes	19.7 [14.8–24.6] 34.2 [25-44.6]	0.0152*	45.5 [33.2–65.5] 64.8 [33.2-105.2]	0.2195

*ASM* aggressive systemic mastocytosis, *CM* cutaneous mastocytosis, *ISM* indolent systemic mastocytosis, *SM-AHN* systemic mastocytosis with associated hematological neoplasm, *SSM* smouldering systemic mastocytosis.

*statistically significant results.

**serum tryptase > 20 µg/L is a diagnostic criterion for SM^14^.

### Comparison of plasma concentration of sclerostin depending bone changes in low-dose computed tomography

To assess the severity of bone disease in 20 patients, low-dose bone computed tomography was used (ASM, *n* = 13; SM-SHN, *n* = 1; SSM, *n* = 3; ISM, *n* = 2; CM, *n* = 1). The CT lesions described and analyzed included the spine, as well as the pelvic and long bones of the lower extremities. Patients with increased sclerosis of spongiosa bone had significantly higher sclerostin levels compared to those without such alterations (medians: 37 vs. 15.2 [pmol/l], respectively) (Table 3).

**Table 3:** Association between sclerostin [pmol/l] and bioactive sclerostin [pmol/l] concentrations and the presence of bone lesions on low-dose computed tomography [*n* = 20].

Variable	Sclerostin [pmol/l]		Bioactive sclerostin [pmol/l]	
	Median [IQR]	*p*	Median [IQR]	*p*
Reconstruction of the spongy bone				
No	24.8 [15.2–37.9]	0.1564	49.5 [35.5–77.8]	0.5389
Yes	37 [25.2–50.1]		64.2 [38.3–92.8]	
Increased sclerosis of spongy bone				
No	15.2 [14.3–16.1]	0.0438*	35.5 [26.8–44.1]	0.1472
Yes	37 [28.1–46.9]		64.2 [42.5-100.8]	
Osteosclerotic lesions				
No	16.1 [16.1–16.1]	0.1934	44.1 [44.1–44.1]	0.5437
Yes	34.7 [23.7–46.9]		63.1 [36.1–96.4]	
The size of the osteoclerotic lesions				
< 1 cm	40.8 [30.7–46.9]	0.3798	69.1 [34-108.3]	0.4943
≥ 1 cm	33.7 [23.5–33.7]		63.1 [37-66.3]	
Osteolytic lesions				
No	31.4 [16.1–46.8]	0.7055	76.2 [44-108.3]	0.6142
Yes	34.2 [22.3–46.9]		59 [34-83.4]	
The size of the osteolytic lesions				
< 1 cm	32.1 [21.2–37.0]	0.2110	43.6 [26.8–73.2]	0.1234
≥ 1 cm	39.3 [22.2–53.2]		65.6 [46.9-118.6]	
Obliteration of the marrow cavities				
No	39.3 [21.9–44.9]	1.0000	44.1 [42.9–92.2]	1.0000
Yes	33.6 [22.1–44.6]		59 [33.2–92.1]	

*Statistically significant results.

### Correlation between sclerostin concentration and demographic and clinical variables

We did not observe a significant correlation between sclerostin concentration nor bioactive sclerostin, and clinical and demographic factors such as age, ECOG, bone marrow infiltration by neoplastic mast cells, or assessed morphological changes in the skeleton (viz. number of osteosclerotic nor osteolytic lesions).

### Correlations between sclerostin concentration and laboratory parameters

**Fig. 3:**
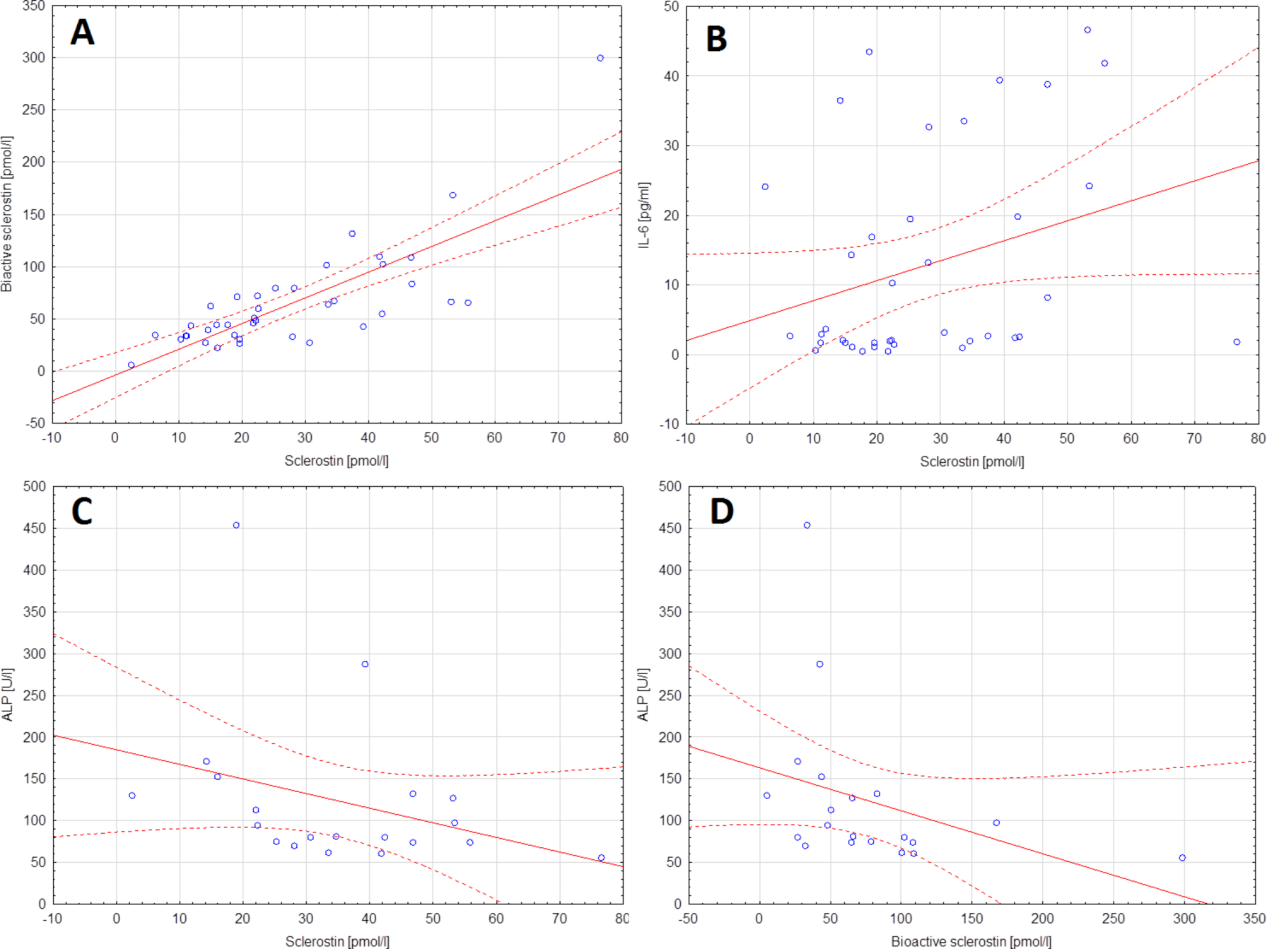
The concentration of sclerostin (mean ± SD)[pmol/l] (**a**) and its bioactive form (mean ± SD)[pmol/l] (**b**) in the supernatants from the HMC1.2 cell line after 6, 24, and 48 h after stimulation with IL-6 compared to the control group - the same cell line not subjected to IL-6 stimulation. * - statistically significant values (*p* < 0.05).

A statistically significant positive, moderate correlation was observed between the concentration sclerostin and its bioactive form (strong correlation; rho = 0.730; *p* < 0.0001; Fig. 3A) and IL-6 (weak correlation; rho = 0.321; *p* = 0.0463; Fig. 3B). On the other hand, negative moderate correlation between ALP activity and sclerostin (rho=-0.458; *p* = 0.0422; Fig. 3C) or its bioactive form (rho=-0.553; *p* = 0.0115; Fig. 3D) was noted.

## Discussion

Sclerostin is secreted by osteocytes to inhibit signaling pathways involved in the activation, proliferation and differentiation of osteoblasts from mesenchymal cells. Expression of the protein is dependent on a number of factors, including mechanical load or hormonal factors, among others. When bone is damaged, osteocytes downregulate sclerostin production and secretion^22^, stimulating bone remodeling mechanisms toward bone formation and repair processes^23^. Sclerostin acts antagonistically to the intracellular Wnt signaling pathway (Wingless signaling) by binding to LRP5 and LRP6 (low-density lipoprotein co-receptors), which are multifunctional parts of the receptor for low-density lipoprotein (LDL)^24^. In addition, it increases osteoclast formation by decreasing the expression of osteoprotegerin (OPG), a “decoy” receptor for receptor activator of nuclear factor kappa-B ligand (RANKL), which increases its expression, enhancing bone resorption^25^.

The *SOST* gene is located on the long arm of chromosome 17. Loss-of-function mutations of the gene cause a condition called sclerosteosis, while insufficient expression is responsible for the pathogenesis of van Buchem disease^26–28^. The excessive bone formation observed in these conditions in the absence or deficiency of sclerostin suggests its suppressive effect on bone formation processes^28,29^.

Sclerostin is involved in the pathogenesis of many bone diseases, although its exact functions are not yet clearly understood. Sclerostin levels are decreased in hyperparathyroidism, after mechanical stress, in estrogen excess and with increased corticosteroids levels^28–33^. Elevated sclerostin levels have been reported in chronic kidney disease, with a significant increase with disease progression, as well as in type 2 diabetes and in the elderly^32–34^. Elevated levels of sclerostin have been reported in osteoporosis - where romosozumab (humanized IgG2 monoclonal antibody that binds to the aforementioned protein) has been successfully used for treatment^35^.

Abnormal activation of the Wnt/β-catenin signaling pathway has been shown to contribute to the development and progression of selected solid tumors and hematologic malignancies^36–41^.

Human metastatic MDA-MB-231 cells inhibit Runx2-dependent osteoblast differentiation through sclerostin, leading to the development of metastatic bone lesions^42^. Zhu et al. showed that breast cancer tissues and breast cancer bone metastases (BCBM) showed positive sclerostin expression (80% and 86.7%, respectively), while results in patients with benign lesions were negative. Moreover, increased sclerostin expression was observed in BCBM compared to localized breast cancer and benign breast tumors^43^.

Sclerostin levels are significantly elevated in patients with prostate cancer. Moreover, Garcia-Fontana et al. showed that patients undergoing androgen deprivation therapy (ADT) had significantly higher sclerostin levels compared to prostate cancer patients without ADT treatment: ADT 64.52 ± 27.21 pmol/l, non-ADT 48.24 ± 15.93 pmol/l, healthy controls 38.48 ± 9.19 pmol/l, *p* < 0.05. The authors found a negative correlation between serum sclerostin and androgen levels (total testosterone: *r*=-0.309, *p* = 0.029; bioavailable testosterone: *r*=-0.280, *p* = 0.049; free testosterone: *r*=-0.299, *p* = 0.035)^44^.

Recently, the gene encoding sclerostin has been shown to be expressed in patients with multiple myeloma, which is associated with the pathogenesis and prognosis of the disease^16,45^. Increased secretion of sclerostin by myeloma cells has been shown to inhibit osteoblast function^46^. Terpos et al. evaluated serum sclerostin levels in 157 patients with newly diagnosed multiple myeloma, 25 patients with relapsed disease and 21 healthy controls. They found higher levels of sclerostin in patients with newly diagnosed multiple myeloma compared to controls (mean: 0.48 ng/ml vs. 0.31 ng/ml; *p* = 0.01). Patients in ISS-3 stage also had higher levels of circulating sclerostin compared to those in ISS-1 stage (mean: 0.71 ng/ml vs. 0.35 ng/ml; *p* = 0.001). The correlation between sclerostin levels and overall survival was also investigated. Patients with sclerostin levels of 0.62 ng/ml or higher had a median survival of 27 months compared to 98 months in the other patients (*p* = 0.031)^47^. Mabile et al. also described a significant increase in sclerostin levels in patients with multiple myeloma 4 months before relapse. The authors suggested the possibility of using sclerostin as a potential early marker of relapse^48^.

There are limited data available from in vitro studies and studies among patients with mastocytosis regarding the function of sclerostin in the pathogenesis of the disease. Elevated serum sclerostin levels have been reported in patients with SM. Rabenhorst et al. compared the levels of bone turnover cytokines, including IL-6, RANKL, OPG, Dkk-1 and SOST, as well as tryptase, calcium, parathormone and vitamin D in the serum of 21 ISM patients with osteopenia or osteoporosis and in healthy volunteers (*n* = 10). In addition, bone marrow biopsies from ISM patients were immunohistochemically stained and evaluated by immunofluorescence, and bone markers were measured in cell culture supernatants from various cell lines. Patients with ISM had significantly higher serum levels of RANKL, OPG and SOST. Moreover, high levels of RANKL, OPG and SOST were detected in the supernatants of the HMC1 cell line, with production of all three cytokines more prominent in HMC1.2, which carries the KIT D816V mutation. These results indicate the involvement of the RANKL/RANK/OPG and Wnt pathways in osteoporosis mediated by mast cells^18^.

Rossini et al. measured serum SOST levels by ELISA (Biomedica Medizinprodukte GmbH & Co. KG, Vienna, Austria) in a group of 22 ISM patients (mean duration of disease: 10.9 ± 9.3 years) with osteopenia or osteoporosis. They found no differences compared to healthy age- and gender-matched subjects (*n* = 50; mean age ± SD, 55 ± 14 years). They confirmed a significant positive correlation between age and SOST levels, as previously reported in other studies^30,49–51^.

In our study, we demonstrated that unstimulated, neopalstic mast cells (HMC-1.2 cell line) are capable of secreting sclerostin and that stimulation with IL-6 (at a concentration of 100ng/ml) results in a significant increase in *SOST* gene expression. This would suggest the potential role of sclerostin and the Wnt pathway as one of the components of the pathomechanism of bone damage in mastocytosis. Moreover, we observed significantly higher sclerostin levels in the plasma of patients diagnosed with more advanced disease stages such as ASM, SM-AHN, and SSM compared to patients with ISM and CM. Significantly higher sclerostin levels were observed in patients with tryptase levels > 20ug/l. We also observed a statistically significant negative, moderate correlation between sclerostin and its bioactive form and the concentration of ALP - a sensitive and reliable marker of osteoblastic activity^52^, and a positive correlation between sclerostin and IL6 - another inhibitor of Wnt-mediated osteogenesis^53^. Based on the previously documented effect of sclerostin on bone formation inhibition^20,54^, bone formation markers might be expected to be correlated inversely with sclerostin levels. On the other hand, there are reports that sclerostin is involved in bone mineralization due to its potential interaction with vitamin D, PTH and FGF-23^14,55^. The exact mechanism of this regulation remains unknown, especially due to the different results of in vitro and in vivo studies^14^. We found no significant correlations between sclerostin or its active form and the level of ALP activity, calcium or phosphorus concentration. Previous researches also showed no evident correlations between sclerostin and the mentioned markers^56^. Furthermore, we did not observe a significant association between bone fractures and the level of sclerostin or its active form. The literature on the relationship between bone fractures and sclerostin levels is limited, and the results presented so far are inconsistent^57–60^. In conclusion, there is a clear need for further studies on larger groups of patients. Assessing the relationship between sclerostin levels and radiological changes in the skeleton using low-dose computed tomography, we observed that in patients with increased sclerosis of the spongy bone, significantly higher sclerostin concentrations are present. However, due to the small (*n* = 20), heterogeneous study group, the obtained results may be inconclusive.

*Limitations of the study.* Our experiment with HMC-1.2 did not include an assessment of the final IL-6 concentration (taking into account the sum of the added IL-6 and that secreted by the tested cell line itself). The study group was relatively small, due to the rarity of the disease in the community. In addition, we did not describe comorbidities in the patients studied, including hormonal and renal disorders. Further studies would therefore require an extension of the study group with the addition of missing clinical data.

## Conclusions

Mast cells in vitro are capable of secretion of sclerostin and its level correlates with some radiological and clinical features of bone disease in SM patients. It suggests the impact of sclerostin on the complex process of bone remodeling in patients with mastocytosis and justify the need for further research using an experimental bone formation model and larger patient groups.

## Data Availability

The data presented in this study are available from the corresponding author upon reasonable request. They are not publicly available due to the fact that the data sheet contains information that exceeds the scope of this study and which may be used for other research papers in the future.
